# Effect of isotretinoin (Netlook) on the testis of adult male albino rats and the role of omega 3 supplementation: A histological and biochemical study

**DOI:** 10.1111/jcmm.17546

**Published:** 2022-09-13

**Authors:** Fatma Al‐Zahraa N. Al‐Shahed, Hala H. Shoeb, Mohammad M. El‐Shawwa

**Affiliations:** ^1^ Department of Histology, Faculty of Medicine for Girls Al‐Azhar University Cairo Egypt; ^2^ Department of Anatomy, Faculty of Medicine for Girls Al‐Azhar University Cairo Egypt; ^3^ Department of Physiology, Faculty of Medicine for Girls Al‐Azhar University Cairo Egypt

**Keywords:** electron microscope, isotretinoin, omega 3, PCNA, PUFAs, testis

## Abstract

Isotretinoin is an oral retinoid which used across the world in the treatment of patients especially adolescents complaining of acne. In spite of the prevalent clinical use of isotretinoin, the generation of oxidative stress with the affection of several organs leads to the limitation and restriction of its use. Omega‐3 (N‐3) is an essential polyunsaturated fatty acid (PUFAs) with powerful antioxidant properties. The aim of this study was to investigate the histological and biochemical changes occurring in the rat testis following isotretinoin intake and to evaluate the role of omega 3 supplementation in ameliorating testicular damage. Thirty adult male albino rats were divided equally into three groups. Group I is the control group, group II received isotretinoin (1.0 mg/kg/day) dissolved in distilled water and group III received isotretinoin (1.0 mg/kg/day) and omega 3 (400 mg/kg/day). Testis samples were collected and processed for light and electron microscopic examination. The blood samples were collected for biochemical assessments. Results indicated that isotretinoin caused histological changes in all stages of spermatogenesis and alterations of the hormonal assay. These changes in the rat testis which were corrected by omega 3 use.

## 1 INTRODUCTION

In patients suffering from acne, topical treatment is effective; however, oral isotretinoin is the first choice in the management of severe and resistant cases of acne leading to long remission or even permanent cure. Acne is a self‐limited chronic inflammatory disease of the pilosebaceous unit, usually affecting the adolescents. The clinical presentation of acne is frequently presented in form of pustules, erythematous papules, and less frequently, nodules or pseudocysts.^1^ Isotretinoin is an oral retinoid (vitamin‐A derivative) that acts through inhibition of the differentiation of sebaceous glands and correction of keratinization defects in the follicle. It is prescribed in other dermatological diseases such as psoriasis, dermatological lesions of systemic lupus erythematosus, ichthyosis and in the prevention of many types of skin cancers.^2^ Food and Drug Administration (FDA) in The United States of America approved isotretinoin in the 1980s with recommended daily dose of (0.5–1.0 mg/kg).^3^ Low‐dose isotretinoin (0.3–0.4 mg/kg/day) shows better efficacy and tolerability.^4^

Omega 3 fatty acids (N‐3polyunsaturated fatty acids) (PUFA) are α‐linolenic acid (ALA), eicosapentaenoic acid (EPA) and docosahexaenoic acid (DHA). They are found naturally in fish oil, flaxseed and some nuts and have anti‐inflammatory effects.^5^ Omega3 supplementation have protective roles in the liver, kidney, cardiovascular system, immune system disorders, psychological status, maternal and offspring health.^6^ N‐3 PUFAs are important ingredients of cell membranes and play a role in the integrity of various membrane channels and receptors.^7^

In this study, omega 3 was used as a protection against the effect of isotretinoin testicular damage which may lead to reproductive dysfunction. To our knowledge, this is the first work to assess the protective effects of omega‐3 against isotretinoin testicular damage.

## 2 MATERIAL AND METHODS

### 2.1 Animals

Thirty adult male albino rats weighting 140–170 g were used in this study. All animal procedures were performed in accordance with the guide for the care and use of laboratory animals and approved by the Animal Ethical Committee at The Faculty of Medicine for Girls, Al‐Azhar University. The principles of laboratory animal care were followed, as well as the specific national laws, when applicable. The examined rats were divided equally into 3 groups:

G I: Control group was kept without medications.

G II: Received isotretinoin at a single oral daily dose of (1.0 mg/kg/day) for 21 successive days according to^8^ then sacrificed. This administrated dose was equivalent to the average human therapeutic dose.

G III: Received isotretinoin (1.0 mg/kg/day) concomitant with omega 3 (400 mg/kg/day) orally for 21 successive days^9^ then sacrificed. This administrated dose was adjusted by using the formula of Paget and Barnes.^10^

By using this formula, the rat dose = the human dose × 18/1000.

Isotretinoin tablet containing 20 mg was dissolved in 20 ml distilled water to produce a suspension containing (1 mg of isotretinoin /ml). Rat dose = The human dose (1 mg) × 18/1000 = 0.018 mg. Each rat received 0.018 ml which contained 0.018 mg isotretinoin.

Each omega 3 soft gelatinous capsule contained 1000 mg omega‐3 fish oil. The capsule was carefully evacuated by 1 ml syringe; then, the content was dissolved in in 9 ml corn oil. So, the 10 ml of oily solvent will contain 1000 mg omega‐3 fish oil. Rat dose = The human dose (400 mg) × 18/1000 = 7.2 mg. The equivalent dose 7.2 mg of omega 3 was administered in 0.72 ml of the oily solvent.

### 2.2 Drugs

Isotretinoin was purchased as a tablet containing 20 mg with a trade name Netlook from Al Andalous For Pharmaceutical Industries. Cairo. Egypt. Omega‐3 PUFAs oil was purchased from South Egypt Drug Industries Company (SEDIC), Cairo, Egypt. It was available in the form of capsules containing (1.0 g) omega‐3 fish oil EPA and DHA.

### 2.3 Biochemical technique

At the end of the experiment, blood samples were collected from 12 to 14 h fasting rats, from retro‐orbital sinus by capillary tubes under light ether anaesthesia. The collected blood samples were centrifuged then sera were separated and stored at −20°C until the time of use. Serum testosterone was assayed by rat testosterone ELISA kit of DRG International, Inc., USA, as previous publication.^11^ Luteinizing hormone (LH) was assayed by rat LH ELISA kit of DRG International, Inc., USA, as mentioned.^12^ Malondialdehyde (MDA) production following the method of^13^ superoxide dismutase (SOD) activity was determined by ELISA as described previously.^14^

### 2.4 Histological techniques

All studied animals were sacrificed by ether inhalation with excision of the two testes. One testis was fixed in Bouin's solution and processed for light microscopic examination^15^ to be stained by Haematoxylin and eosin stain (H&E) and Periodic acid Schiff's (PAS). In addition, proliferating cell nuclear antigen (PCNA) immunohistochemical techniques were used for detection of active cell proliferation in testicular tissue.^16^ The other testis was fixed in glutaraldehyde and processed for electron microscopic (E M) examination.^17^

### 2.5 Morphometrical measurements

It was carried out using a computerized image analysis system (Software Leica Quin 500). The basement membrane thickness, epithelial height, seminiferous tubular diameter and the mean area % of PCNA‐positive cells were measured in 10 non‐overlapping fields per group using a magnification (400×).

### 2.6 Statistical analysis

Statistical Analysis was conducted using (SPSS) program version 22, data expressed as mean ± SD for statistical analysis, using analysis of variance [anova] test to compare between the different studied groups. The value of significance was taken at (*p*‐value ≤0.05).

## 3 RESULTS

### 3.1 A: Biochemical results

Administration of isotretinoin to rats (G II) caused significant decrease in serum testosterone and SOD and significant increase in LH and MDA if compared to control group (G 1). Co‐administration of isotretinoin and omega 3 to rats (G III) caused a significant increase in serum testosterone and SOD and a significant decrease in LH and MDA if compared to G II, but these parameters still significantly changed if compared to G I (Table 1).

**TABLE 1:** Changes of serum testosterone, LH, MDA and SOD levels in different experimental groups

	Groups				
Parameters	G1*N* = 6	G2*N* = 6	G3*N* = 6	Test of sig.	*p*‐value
Testosterone (ng/ml ± SD)	1.54 ± 0.3	0.6 ± 0.2^a^	1.1 ± 0.1^a^ ^,^ ^b^	anova *F* = 30	0.000*
LH (ng/ml ± SD)	2.77 ± 0.5	6.9 ± 2.2^a^	4.31 ± 0.9^a^ ^,^ ^b^	anova *F* = 13	0.001*
MDA (nmol/gm ± SD)	8.15 ± 0.9	28.11 ± 1.2^a^	13.54 ± 0.9^a^ ^,^ ^b^	anova *F* = 615	0.000*
SOD (u/gm ± SD)	3.1 ± 0.02	0.9 ± 0.1^a^	2.1 ± 0.3^a^ ^,^ ^b^	anova *F* = 359	0.000*

^a^ Significant values versus group 1.

^b^ Significant values versus group 2.

The Significance of * values indictes *p*‐value ≤ 0.05.

### 3.2 B: Histological results

**FIGURE 1:**
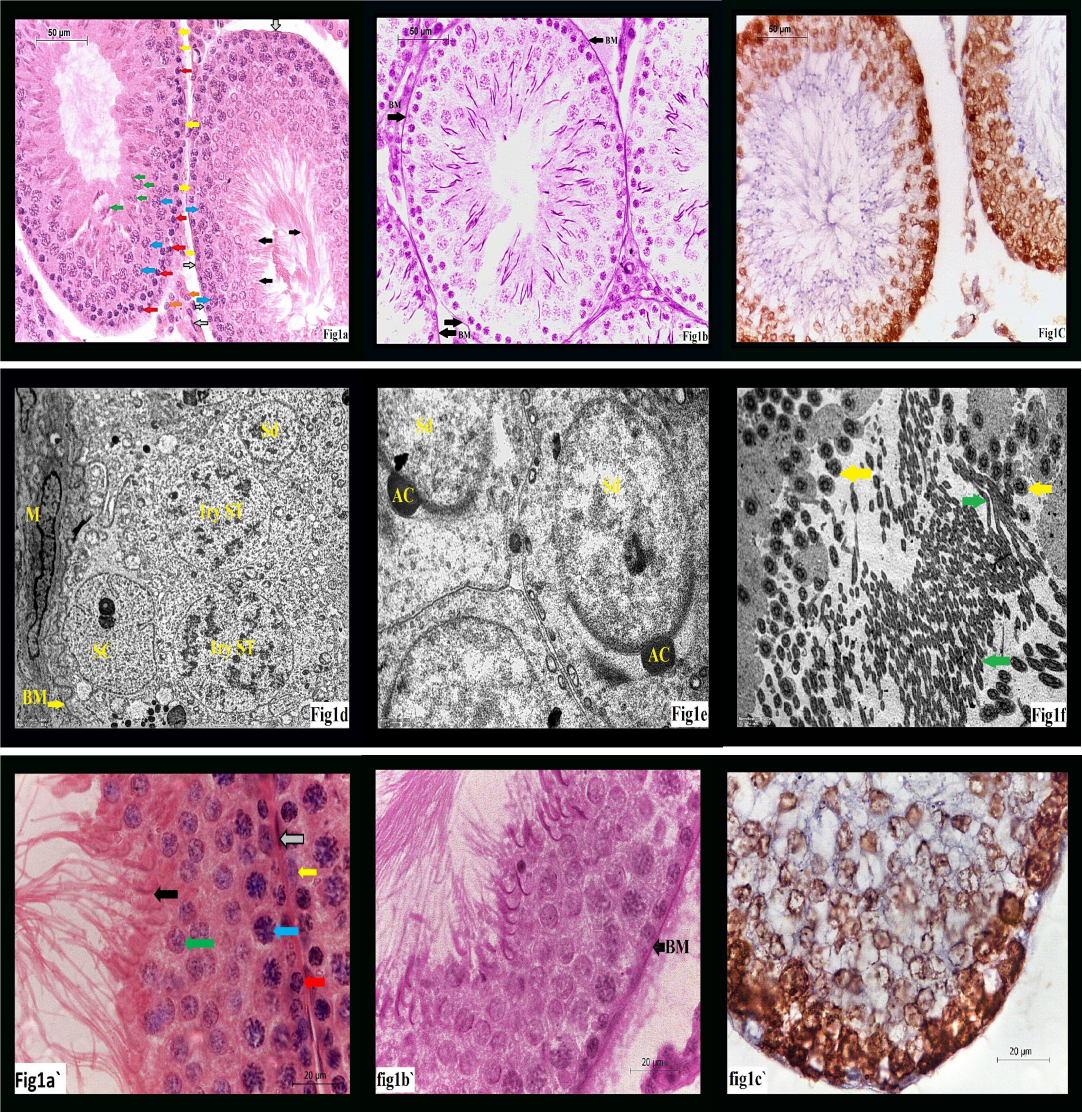
(A) A photomicrograph of GI showing: Sertoli cell (yellow arrow), spermatogonia (red arrow), primary spermatocyte (blue arrow), spermatid (green arrow), sperm (black arrow), Leydig cell (orange arrow) and myoid cell (grey arrow). (H&E ×400). (B) A photomicrograph of GI showing: normal thickening of basement membrane (BM) of seminiferous tubules. (PAS ×400). (C) A photomicrograph of GI showing: normal PCNA staining of tubular cells & interstitial cells. (PCNA ×400). (D) An electron micrograph of G1 showing: Sertoli cell (SC), 1ry spermatocytes (1ry ST), spermatid (Sd), myoid cell (M) and basement membrane (BM). (Uranyl acetate & Lead citrate ×4000). (E) An electron micrograph of G1 showing: spermatid (Sd), acrosomal cap (AC). (Uranyle acetate & lead citrate ×8000). (F) An electron micrograph of G1 showing: normal amount and structure of sperms both in longitudinal (green arrow) and transverse sections (yellow arrow). (Uranyle acetate & lead citrate ×3000). (A') showing higher magnification for A (H&E ×1000). (B') showing higher magnification for B (PAS ×1000). (C') showing higher magnification for C (PCNA ×1000)

Examination of H&E‐stained section of control group revealed that the testis was formed of groups of seminiferous tubules with regular outlines and inter‐tubular tissue in between. Each seminiferous tubule was lined by 4–6 layers of germinal epithelium at different stages of spermatogenesis and Sertoli cells, with its pyramidal nuclei all resting on thin basement membrane. The germinal epithelium was composed of spermatogonia, primary spermatocyte, rounded and elongated spermatids, and mature sperms. The flagella of mature sperms were seen in the lumen of the tubules. (Figure 1A).

**FIGURE 2:**
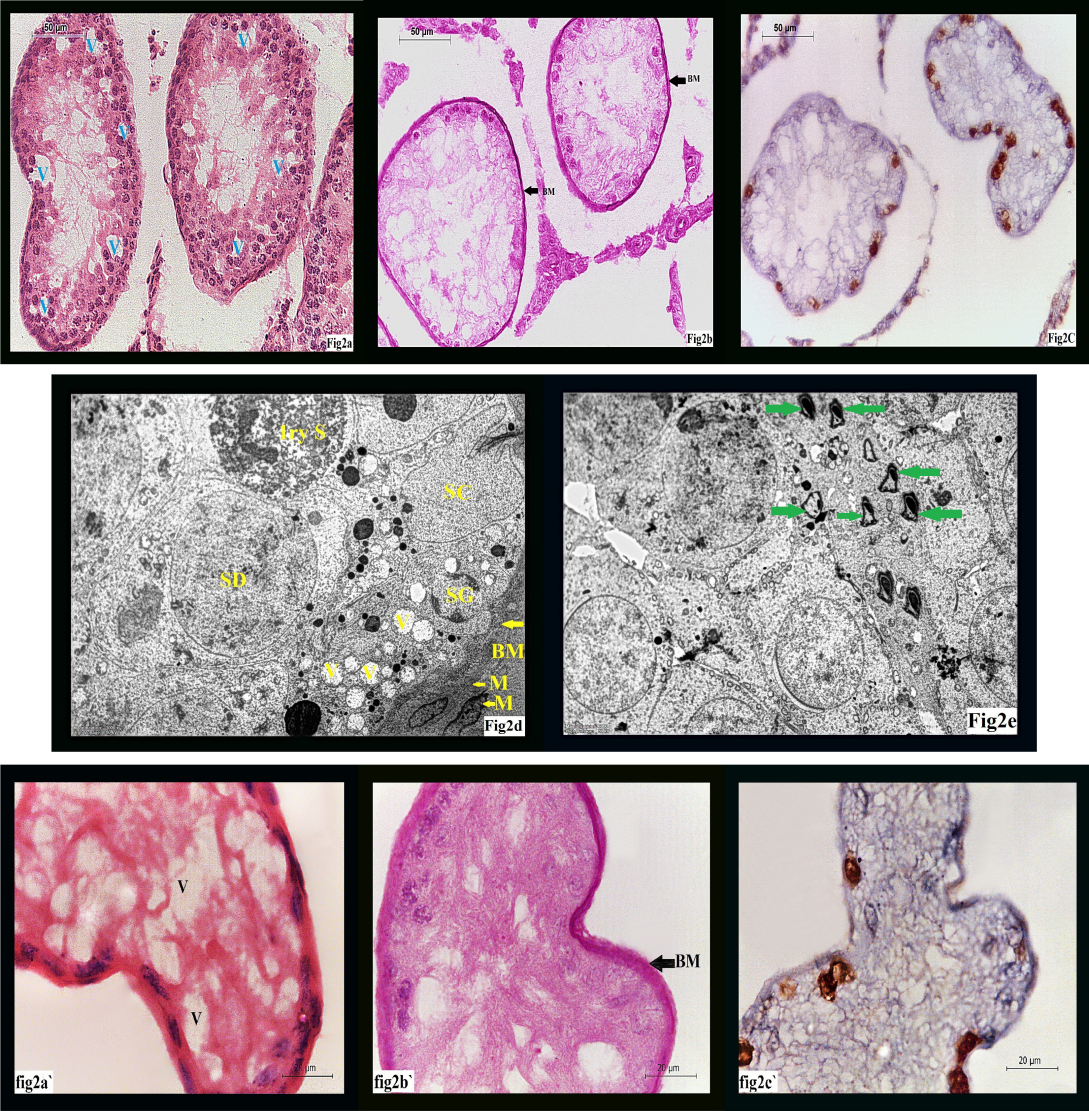
(A) A photomicrograph of GII showing: decreased tubular size, decreased epithelial height and multiple vacuoles (V). (H&E ×400). (B) A photomicrograph of GII showing: increased thickening of basement membrane (BM) of seminiferous tubules. (PAS ×400). (C) A photomicrograph of GII showing: decreased PCNA staining of tubular cells & interstitial cells. (PCNA ×400). (D) An electron micrograph of GII showing: multiple vacuoles (V), thickened basement membrane (BM), Sertoli cell (SC), 1ry spermatocytes (1ry ST), spermatid (Sd) and spermatogonia (SG). (Uranyl acetate & Lead citrate ×3000). (E) An electron micrograph of GII showing: abnormal forms of sperms (green arrows). (Uranyl acetate & Lead citrate ×3000). (A') showing higher magnification for A (H&E ×1000). (B') showing higher magnification for B (PAS ×1000). (C') showing higher magnification for C (PCNA ×1000)

Examination of H&E‐stained sections of G II group showed irregular tubular outlines together with decreased tubular diameter, increased inter‐tubular distance, decreased germinal epithelial height, few remaining apoptotic germ cells, disappearance of sperms from the lumen, most of nuclei were small darkly stained pyknotic nuclei, and inter‐cellular vacuolation (Figure 2A).

**FIGURE 3:**
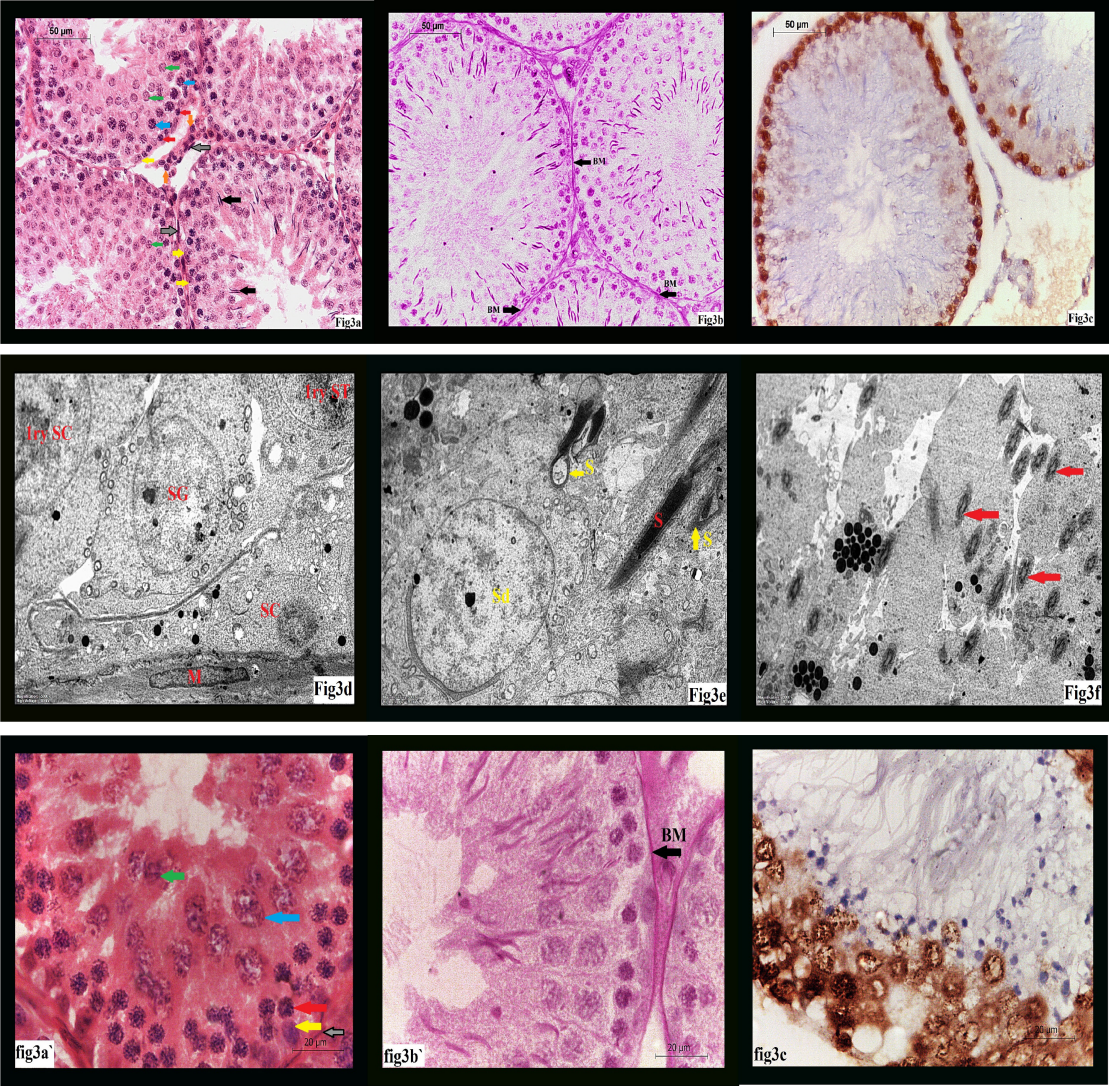
(A) A photomicrograph of GIII showing: nearly normal tubular size, epithelial height, and cells Sertoli cell (yellow arrow), spermatogonia (red arrow), primary spermatocyte (blue arrow), spermatid (green arrow), sperm (black arrow), Leydig cell (orange arrow) and myoid cell (grey arrow). (H&E ×400). (B) A photomicrograph of GIII showing: nearly normal thickness of basement membrane (BM) of seminiferous tubules. (PAS ×400). (C) A photomicrograph of GIII showing: increased PCNA staining of tubular cells in relation to GII. (PCNA ×400). (D) An electron micrograph of GIII showing: Sertoli cell (SC), 1ry spermatocytes (1ry ST), spermatogonia (SG) and myoid cell (B). (Uranyl acetate & Lead citrate ×5000). (E) An electron micrograph of GIII showing: some normal sperms (red arrow), some abnormal sperms (yellow arrow) and spermatid (Sd). (Uranyl acetate & Lead citrate ×5000). (F) An electron micrograph of GIII showing: increased amount of sperms (red arrows) compared to GII. (Uranyl acetate & Lead citrate ×5000). (A') showing higher magnification for A (H&E ×1000). (B') showing higher magnification for B (PAS ×1000). (C') showing higher magnification for C (PCNA ×1000)

Examination of H&E sections of group III showed improvement of tubular structure nearly like the control group. Increased regular tubular diameter with increased epithelial height was noticed with re‐appearance of sperms with its characteristic flagella. Decreased epithelial vacuolation and decreased inter‐tubular distance were also noticed. (Figure 3A).

PAS‐stained section of control group showed thin regular basement membrane surrounding normal seminiferous tubules. (Figure 1B) while examination of PAS section of group II revealed thick irregular basement membrane. (Figure 2B). After treatment with omega III, group III sections stained with PAS showed nearly normal regular thickness of basement membrane compared to control group (Figure 3B).

PCNA staining of control group showed PCNA‐positive reaction mainly in spermatogonia and primary spermatocytes (Figure 1C), while marked decrease in PCNA‐positive (PCNA +ve cells) was noticed in group II (Figure 2C). In group III, there was marked increase (PCNA +ve cells) (Figure 3C).

In electron microscopic examination of control group GI revealed basement membrane of seminiferous tubules, myoid cell also appeared as elongated cell on the basement membrane from outside; also, spermatogonia, primary spermatocytes, spermatids and spermatogonia were seen. (Figure 1D–F).

Giving isotretinoin to rats of GII revealed vacuolation and apoptosis of most of the cells, thickening of basement membrane, abnormal forms of sperms and apparently decreased number of spermatozoa (Figure 2D,E).

Application of omega 3 together with isotretinoin in GIII caused improvement of vacuolation, cell structure, basement membrane thickness, structure and number of spermatozoa. (Figure 3D–F).

### 3.3 C. Morphometric results

1. Administration of isotretinoin to (G II) caused significant increase in basement membrane thickness and decrease in per cent of PCNA +ve cells compared to the control group (G I). In (G III), Co‐administration of isotretinoin and omega 3 caused significant decrease in basement membrane thickness and increase in the percentage of PCNA +ve cells compared to G II (Table 2 and histogram II a, b).

**TABLE 2:** Comparison of basement membrane thickness and per cent of PCNA +ve cells among all the experimental group

	Studied groups				
Items	G1*N* = 6	G2*N* = 6	G3*N* = 6	Test of sig.	*p*‐value
BM (Mm ± SD)	1.29 ± 0.1	1.89 ± 0.2	1.28 ± 0.1	anova *F* = 32	0.001*
% PCNA(±SD)	25.35 ± 6.2	15.51 ± 3.8	18.96 ± 4.5	anova *F* = 6	0.011*

*Note*: G2 significant with G1&G3.

The Significance of * values indictes *p*‐value ≤ 0.05.

2. Administration of isotretinoin to (G II) caused a significant decrease in tubular diameter and epithelial height compared to the control group (G I). In (G III), co‐administration of isotretinoin and omega 3 caused a significant increase in epithelial height and tubular diameter compared to G II (Table 3 and histogram 3).

**TABLE 3:** Comparison of changes in seminiferous tubular diameter and epithelial height among all the experimental group

	Studied groups				
Items	G1*N* = 6	G2*N* = 6	G3*N* = 6	Test of sig.	*p*‐value
TD (Mm ± SD)	291.3 ± 2.6	197.2 ± 2.3	271.6 ± 2.6	anova *F* = 2366	0.000*
EH (Mm ± SD)	97.1 ± 1.6	70.6 ± 1.5	89.4 ± 1.7	anova *F* = 433	0.000*

*Note*: G1 significant with G2 & G3.

G2 significant with G1 & G3.

G3 significant with G1 & G2.

The Significance of * values indictes *p*‐value ≤ 0.05.

## 4 DISCUSSION

Testing medicines to determine the possible harmful health effects and exposure levels that can be considered safe is of utmost importance. Most adverse effects involving the use of retinoids, including isotretinoin, are related to the skin and mucous membranes, nervous system, skeletal muscle, haematopoietic, lymphatic, gastrointestinal, cardiorespiratory and genitourinary systems.^18^, ^19^

In this study, we used biochemical, histopathological and immunohistochemical methods to detect the effects of isotretinoin on rats' testis. Using light microscopy, isotretinoin caused irregular seminiferous tubules with thick irregular basement membrane. The seminiferous tubules were lined by few apoptotic spermatogenic cells with cytoplasmic vacuolation associated and diminished spermatozoa. Similar observations were found with reduction in testicular mass associated with lesions in the seminiferous epithelium and Leydig cells, which might affect the spermatogenesis.^19^ These histological changes in the current study were confirmed by significant decrease in serum testosterone and significant increase in LH levels. The same results were reported with a significant decrease in serum testosterone and a significant increase in serum LH level with isotretinoin treatment.^20^ The increase in LH level might be explained by the negative feedback effect from reduced testosterone level.^21^ This reduction might be mediated via the gonads which contained abundant retinoid receptors.^22^

Reduction in androgen, testosterone and rostenedione levels were reported after 12 weeks from isotretinoin use.^23^ These effects were mediated via presence of retinoid receptors in tests.^24^ Reduced testosterone levels may explain the effectiveness of isotretinoin in acne treatment. Pathogenesis of acne is depending on level of androgen which directly related to enhancement of sebum production and follicular keratosis.^25^

By electron microscopy, there were abnormal forms of sperms, cytoplasmic vacuolation, apoptosis, thick basement membrane and an apparent decrease in the number of spermatozoa. These changes might be explained by the abnormal process of spermatogenesis due to abnormal testosterone level.^26^

Isotretinoin testicular damage might occur due to the action of retinoic acid on both Sertoli cells and germinal cells pushing undifferentiated spermatogonia into differentiation. However, the change in retinoic acid level might cause alterations in the differentiation of spermatogonia either at the beginning of meiosis or at the beginning of the cycle of the seminiferous epithelium.^27^ Retinoic acid was a potent endocrinologic regulator of the testicular development and germ cell proliferation.^22^ Vitamin A is an essential factor during spermatogenesis.^19^

Vitamin A deficiency could result in disturbance of testosterone secretion, meiotic germ cells degeneration and cessation of spermatogenesis.^24^ However, hypervitaminosis A could lead to disturbance of spermatogenesis rhythm, and retinoid deficiency or excess may be harmful to spermatogenesis and steroidogenesis.^19^

In contrary to these results, systemic isotretinoin caused no change in total testosterone or LH levels with positive effects on all the parameters of the spermiogram.^28^

Furthermore, the positive effect of retinoic acid was dose‐dependent, and with higher doses, signs of hypervitaminosis and degeneration of the spermatocytes were noticed. So isotretinoin effect on sex hormones is time and dose dependent.^29^

In this study, a notable elevation of MDA (representing massive lipid peroxidation) and significant decrease in SOD level with isotretinoin use may reflect increased cellular oxidative stress, which may be another factor in isotretinoin testicular damage. This finding was supported by mitochondrial changes observed by EM. Accordingly, mitochondrial reductive stress might be a possible source of free radicals with the accumulation of reducing equivalents in the mitochondrial electron transport chain, which enhance the production of reactive oxygen species (ROS).^30^, ^31^

In addition, mitochondria may become an oxidative target resulting in lipid peroxidation, DNA cleavage and impaired ATP production.^31^, ^32^ This factor was explained by other studies which clarified that isotretinoin treatment resulted in loss of biological membrane fluidity, neutrophil activation and lipid peroxidation with production of ROS.^33^, ^34^

Regarding level of SOD; which is an indicator of antioxidant activity as it directed the superoxide anion to form hydrogen peroxide.^35^ In this study, the decreased SOD activity may reflect accumulation of superoxide radicals and hydrogen peroxide resulting in a state of oxidative stress. As mentioned before the steroidogenic capacity and proliferation of Leydig cells were deteriorated and apoptosis occur with exposure to oxidants.^36^ Furthermore, ROS induced lipid peroxidation which resulted in changes in testicular functions.^37^

In this study, co‐administration of omega 3 and isotretinoin caused improvement of histological changes caused by isotretinoin nearly to be similar to the control group G 1. These findings could be explained by the high amounts of PUFA in the composition of the male germ cell membranes, which are mainly susceptible to lipid peroxidation.^38^

Due to the high content of DHA in omega‐3, it is capable to restore fertility through the protection of the germ cells, spermatozoa and spermatids membranes architecture.^39^

These findings were similar to other studies which found that fish oil in obese male mice could improve sexual hormones synthesis by increasing the number of Leydig cells and diminishing apoptosis then stimulating the testosterone biosynthesis through the adenyl cyclase, cyclic AMP, protein kinase A and splicing factor‐1 pathways.^40^

Our data showed that compared to isotretinoin alone, the treatment of rats with omega 3 and isotretinoin increases levels of antioxidants enzymes (SOD) and reduced (MDA).

The administration of omega 3 to male rats caused a significant increase in glutathione level and a significant decrease in MDA level.^41^ Therefore, PUFAs oil improves antioxidant activity and decreased the oxidative stress, so diet rich in omega‐3 can raise the SOD level.^42^ Diet enriched with omega‐3 from fish oil and linseed oil decreases serum cholesterol, triglycerides level and enhances antioxidative status.^43^ In addition, incorporation of PUFAs in cell membrane may reduce the susceptibility of cells to lipid peroxidation, change the fluidity of cell membrane, elevate enzymes activity, enhance receptor function and influence the production of lipid mediator.^44^

This may be associated with the reaction between free radical and PUFAs resulting in avoiding free radical conjugating with fatty acids of the membrane.^45^ ROS had been concerned with reduced fertility and high levels of nitric oxide, which is related to ROS generation.^46^ In this study, PCNA‐positive cells were detected in control rats in spermatogonia and early stage of spermatocytes. However, the number of PCNA‐positive cells was significantly lowered in group GII with isotretinoin use. The reduction in PCNA in testicular germ cells might be as a result of the decrease in proliferative activity and spermatogenesis. This observation is in agreement with previous studies^47^, ^48^ who mentioned that PCNA is an intranuclear polypeptide which involved in DNA replication, excision and repair. They explained that decrease of PCNA‐positive cells in their studies by DNA damage which appeared to be the main cause of decreased number of sperms since spermatozoa require intact DNA during fertilization process.

Lipid peroxidation in the presence of elevated ROS seminal levels and oxidative stress induces DNA damage in both the mitochondrial and nuclear genomes which also play a role in the vulnerability of spermatozoa to damage by the free radicals.^49^

Also, in this study, there was an increase in the of PCNA‐positive cells in testis tissues with omega‐3 use in G III indicating effective protection against the oxidative effects of isotretinoin on the testes. The same observation was noticed when pre‐treatment with fish omega‐3 fatty acids effectively protected testis against the oxidative and apoptotic effects of doxorubicin.^50^

Finally, omega‐3 fatty acids may help clinically in acne cases treated with isotretinoin in different ways. Aside from being an anti‐inflammatory, omega 3 also aids help in treatment of many dermatological problems for example atopic dermatitis, psoriasis and blepharitis.^51^

## AUTHOR CONTRIBUTIONS

**Fatma Al‐Zahraa Nabil Al‐Shahed:** Funding acquisition (equal); methodology (equal); resources (equal); software (equal); supervision (equal); writing – original draft (lead); writing – review and editing (lead). **Hala Hamed Shoeb:** Data curation (equal); funding acquisition (equal); resources (equal); writing – review and editing (equal). **Mohammad Mohammad Elshawwa:** Funding acquisition (equal); investigation (equal); methodology (equal).

## CONFLICT OF INTEREST

The authors confirm that there are no conflicts of interest.

## Data Availability

The data that support the findings of this study are openly available in [repository name] at [fatmanabil.medg@azhar.edu.eg.], reference number [fatmanabil.medg@azhar.edu.eg.].
